# Circulating MicroRNA Biomarkers for Lung Cancer Detection in East Asian Populations

**DOI:** 10.3390/cancers11030415

**Published:** 2019-03-23

**Authors:** Haixin Yu, Zhong Guan, Katarina Cuk, Yan Zhang, Hermann Brenner

**Affiliations:** 1 Division of Clinical Epidemiology and Aging Research, German Cancer Research Center (DKFZ), 69120 Heidelberg, Germany; haixin.yu@dkfz-heidelberg.de (H.Y.); z.guan@dkfz-heidelberg.de (Z.G.); katarina_cuk2@yahoo.de (K.C.); y.zhang@dkfz-heidelberg.de (Y.Z.); 2 Medical Faculty Heidelberg, University of Heidelberg, 69120 Heidelberg, Germany; 3 German Cancer Consortium (DKTK), German Cancer Research Center (DKFZ), 69120 Heidelberg, Germany; 4 Division of Preventive Oncology, German Cancer Research Center (DKFZ) and National Center for Tumor Diseases (NCT), 69120 Heidelberg, Germany

**Keywords:** miRNA, lung cancer, early detection, East Asian populations

## Abstract

Background: Lung cancer (LC) is the leading cause of cancer-related death in Eastern Asia. The prognosis of LC highly depends on tumor stages and early detection could substantially reduce LC mortality. Accumulating evidence suggested that circulating miRNAs in plasma or serum may have applications in early LC detection. We thus conducted a systematic literature review on the diagnostic value of miRNAs markers for LC in East Asian populations. Methods: PubMed and ISI Web of Knowledge were searched to retrieve relevant articles published up to 17 September 2018. Information on study design, population characteristics, investigated miRNAs and diagnostic accuracy (including sensitivity, specificity and area under the curve (AUC)) were independently extracted by two reviewers. Results: Overall, 46 studies that evaluated a total of 88 miRNA markers for LC diagnosis in East Asian populations were identified. Sixteen of the 46 studies have incorporated individual miRNA markers as panels (with 2–20 markers). Three promising miRNA panels with ≥90% sensitivity and ≥90% specificity were discovered, two of which were externally validated. Diagnostic performance of circulating miRNAs in East Asian populations was comparable to previously summarized performance in Western populations. Forty-four miRNAs were reported in both populations. No major differences in diagnostic performance by ethnicity of the same miRNA was observed. Conclusions: Circulating miRNAs or miRNA panels, possibly in combination with other promising molecular markers including epigenetic and genetic markers, may be promising candidates for noninvasive LC early detection. However, large studies with samples collected prospectively in true screening settings are required to validate the promising markers or marker panels.

## 1. Introduction

Lung cancer (LC) is the leading cause of cancer mortality in Eastern Asia, with 950,015 cases and 815,635 deaths estimated in 2018 [1]. Although advances in therapy have led to improvements in survival of LC patients [2], the 5-year survival rate remains very low, mainly due to late diagnosis of disease [3]. Traditional screening methods such as chest radiography and sputum cytology have limited clinical applications as they display low sensitivity and specificity in detection of LC [4]. In recent years, Low-dose CT has been recommended for LC screening in high-risk smokers. However, potential hazards of CT screening, including radiation exposure, high false-positive rates, overdiagnosis and high cost, raise concerns [5,6]. Therefore, in order to reduce LC mortality, effective methods for early diagnosis of LC remain desirable.

MicroRNAs (miRNAs) are a class of single stranded RNAs composed of 18–22 nucleotides, which are involved in the regulation of gene transcription but have no protein coding function, and are widely present in eukaryotic cells [7]. At present, several studies have identified abnormally expressed miRNA patterns in blood specimens of LC patients, such as plasma, serum and exosome, suggesting that circulating miRNA may be useful for LC diagnosis [8,9,10].

Because of the genetic diversity of miRNA expression profiles of populations [11], we previously summarized the diagnostic performance of circulating miRNAs restricted to Western populations [12]. Herein, we systematically reviewed their diagnostic performance in East Asian populations, paying particular attention to the potential differences between the East Asian and Western populations.

## 2. Methods

This systematic review was conducted in accordance with PRISMA recommendations [13].

### 2.1. Literature Search

A systematic literature search was carried out to identify all studies that evaluated circulating miRNAs related to LC. We searched the PubMed and ISI Web of Science databases for relevant articles from inception to 17 September, 2018, using the following keyword combinations: ((lung OR pulmonary) AND (cancer OR carcinoma OR neoplasm OR tumor OR adenocarcinoma OR squamous carcinoma OR malignancy) AND (microRNA* OR miRNA* OR miR* OR let-7*) AND (detection OR diagnosis OR biomarker OR marker) AND (blood OR serum OR plasma)). Duplicate articles were removed.

### 2.2. Eligibility Criteria

**Figure 1:**
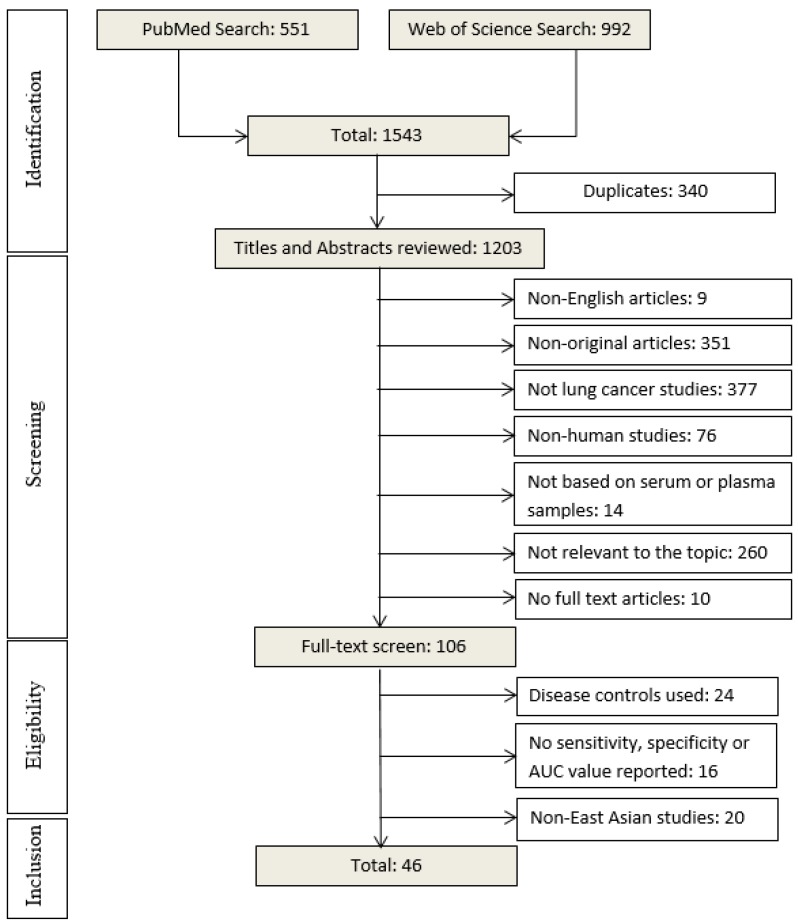
Overview of the literature search process (until 17 September 2018).

Based on the reading of titles and abstracts, we excluded: (1) non-English articles; (2) non-original articles; (3) not lung cancer studies; (4) non-human studies; (5) studies not based on plasma/serum samples; (6) studies not relevant to the topic; (7) no full text articles (Figure 1). The second round of screening was performed by reading full text of articles. In this step, the following studies were excluded: (1) studies using disease controls; (2) studies not reporting key data on diagnostic performance of miRNA markers (such as simple size, sensitivity, specificity, or area under the curve (AUC); (3) Non-East Asian studies.

### 2.3. Data Extraction and Statistical Analysis

Two reviewers (H.Y. and Z.G.) independently read and extracted data from the studies that complied with the inclusion and exclusion criteria described above. From each study, we extracted available data on first author, publication year, country, study design, basic population characteristics (including size, age and sex distribution, and histological type and tumor stage for cases), type of bio-specimen, miRNA measurement method, targeted miRNA markers and diagnostic performance related indicators (including sensitivity, specificity, AUC and *p*-value). Individual miRNAs with *p*-value > 0.05 were dropped. Mean or median age and male proportion of included studies were calculated by statistical software R (version 3.3.3, R Foundation, Vienna, Austria) if this information was not reported but raw data were available. Different name versions of the same miRNAs were incorporated according to the accession number of miRBase provided (http://www.mirbase.org/). Any disagreements were discussed and resolved among the authors.

### 2.4. Quality Assessment

We employed QUADAS-2, an updated and widely recommended tool for quality assessment of diagnostic accuracy studies, to assess the quality of each included study. Four key domains of QUADAS-2 were assessed, i.e., patient selection, index test, reference standard and flow and timing. Each domain is evaluated for risk of bias and the first three domains are evaluated for applicability. QUADAS-2 assessment was conducted utilizing the software Review Manager (version 5.3.5, Cochrane Collaboration, Copenhagen, Denmark).

## 3. Results

### 3.1. Literature Search Result

Our literature search initially yielded 1543 articles using the search terms mentioned above, 551 from PubMed and 992 from Web of Science (Figure 1). After removal of 340 duplicate articles, titles and abstracts of 1203 articles were carefully reviewed. A total of 1097 articles were excluded according to the above described exclusion criteria. The remaining 106 articles were selected for full text reading, of which 60 articles were removed: 24 using disease controls, 16 not reporting sensitivity, specificity or AUC values, and 20 reporting studies in non-East Asian countries. In the end, 46 studies that evaluated diagnostic performance of circulating miRNAs for LC in East Asian populations and published between 2011 and 2018 (Table 1 and Table 2) were eligible for this systematic review [8,9,10,14,15,16,17,18,19,20,21,22,23,24,25,26,27,28,29,30,31,32,33,34,35,36,37,38,39,40,41,42,43,44,45,46,47,48,49,50,51,52,53,54,55,56]. Of the included 46 studies, 45 were from China and one was from Japan [29].

### 3.2. Study Quality and Characteristics

QUADAS-2 assessment was completed by two reviewers (H.Y. and Z.G.) independently. Any inconsistencies were discussed and resolved between the investigators. High applicability concerns were found in the patient selection domain in 10 (22%) of the included studies. Unclear risk of bias were found in the patient selection domain and the index test domain in 24 (52%) and 6 (13%) of the included studies, respectively. No risk of bias or applicability concern was found in the reference standard domain, and the flow and timing domain. Details of QUADAS-2 results of the 46 studies are displayed in Figures S1 and S2.

All 46 studies were case-control studies in which blood samples were collected after disease diagnosis. Of the 46 studies, 43 evaluated individual miRNAs, eight of which conducted independent validation (Table 1). Sixteen studies assessed miRNA panels, 13 of which carried out independent validation (Table 2). Detailed information on each study, including the number of cases and controls, mean or median age, male proportion, specimen type, histological subtype, tumor stage, and diagnostic indicators, are summarized in Table 1 and Table 2. Table 1 additionally shows the p-value for testing the difference of each individual miRNA between cases and controls or the statistical significance of AUC values (indicated in the footnotes of Table 1).

The median numbers (range) of LC cases and controls were 94 (11–514) and 60 (11–360), respectively. Fifteen studies examined miRNAs in plasma [10,16,19,21,27,31,37,41,42,43,48,50,55,56,57], 30 in serum [9,14,15,17,18,20,23,24,25,26,28,29,30,32,33,34,35,36,38,39,40,44,45,46,47,49,51,52,53,54] and one in exosome [8]. Overall, 46 studies evaluated 69 miRNA markers and 19 miRNA panels (in total 88 miRNAs). All 46 studies quantified miRNA levels using quantitative real-time polymerase chain reaction (qRT-PCR), the most commonly used method for miRNA detection over the past 5 years. Only one study conducted by Fan et al. [33] also used fluorescence quantum dots liquid bead array to quantify miRNA levels.

### 3.3. Diagnostic Performance of miRNA Markers

**Figure 2:**
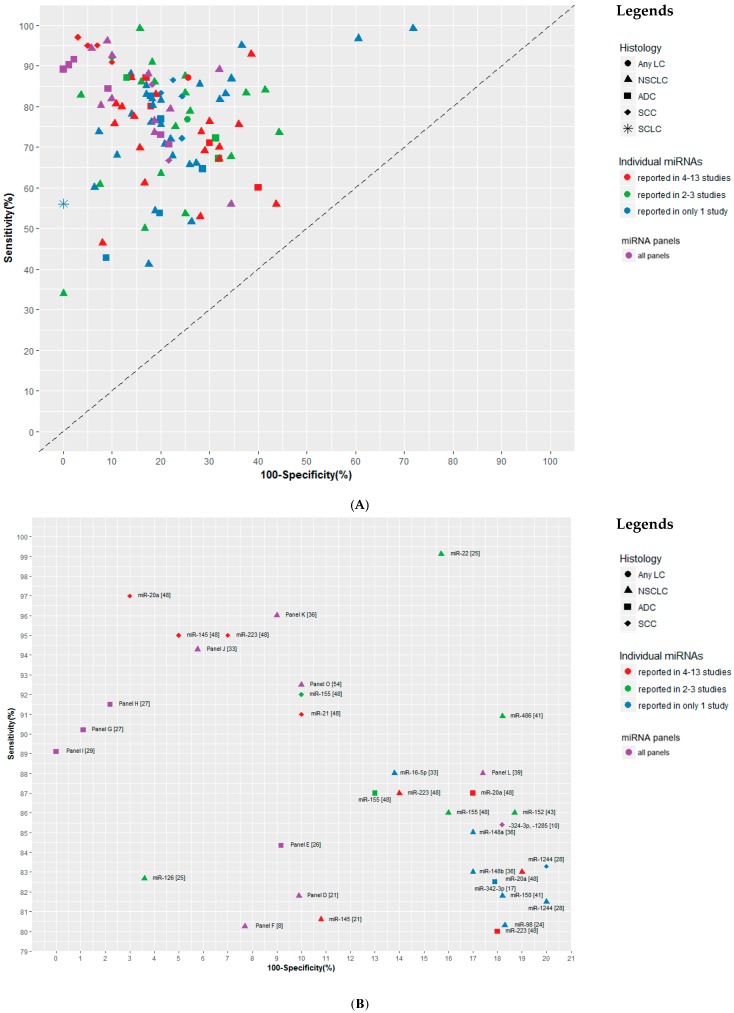
Graphical representation of sensitivity versus specificity of analyzed miRNAs. Sensitivity is plotted on the y-axis while on the x-axis the false positive rate is presented (100-Specificity). (**A**) Overview of all analyzed miRNAs and miRNA panels and (**B**) more detailed representation of miRNAs and miRNA panels with ≥80% sensitivity and ≥80% specificity. Panel D: -145, -20a, -21, -223; Panel E: -146a, -222, -223; Panel F: let-7b-5p, let-7e-5p, -24-5p, -21-5p; Panel G: -628-3p, -339-3p, -425-3p, -532; Panel H: -628-3p, -425-3p, -532; Panel I (20 miRs): -451, -1290. -636, -30c, -22-3p, -19b, -486-5p, -20b, -93, -34b, -185, -126-5p, -93-3p, -1274a, -142-5p, -628-5p, -486-3p, -425, -645, -24; Panel J: -20a-5p, -16-5p, -15b-5p; Panel K: -148a, -148b, -152, -21; Panel L: -25, -125a-5p, -126; Panel O (10 miRs): -20a, -24, -25, -145, -152, -199a-5p, -221, -222, -223, -320. Abbreviations: LC: lung cancer; NSCLC: non-small cell lung cancer; ADC: adenocarcinoma; SCC: squamous cell carcinoma; SCLC: small cell lung cancer.

Of the 88 circulating miRNAs included in the 46 studies, 22 miRNAs were reported in ≥ 2 studies (Table 3). Most identified miRNAs were also included in panels, and only 31 miRNAs were not part of any panel (Table S1). The smallest panel included only two miRNAs [10,34,50], and the largest panel included 20 miRNAs [29]. An overview of the diagnostic performance of all reported miRNAs and miRNA panels is shown in Figure 2A. For individual miRNAs, the median (range) sensitivity and specificity were 76.9% (33.9–99.2%) and 80% (28.2–100%) respectively. The median (range) sensitivity and specificity of miRNA panels were 76.3% (55.9–96%) and 79.6% (65.6–100%) respectively. A more detailed representation of miRNAs and miRNA panels with ≥80% sensitivity and ≥80% specificity is given in Figure 2B (17 individual miRNAs and 11 miRNA panels). Even though several individual miRNAs, such as miR-16-5p [33] and miR-223 [48] showed comparable performance as some of the panels, such as a 3-miRNA panel in Wang’s study [39], miRNA panels generally outperformed individual miRNAs for non-small cell lung cancer (NSCLC), with three miRNA panels showing ≥90% sensitivity and ≥90% specificity (Figure 2B). Two of the three panels were also externally validated [33,54]. Two of 46 studies included LC cases of any histological subtypes [37,52], 34 studies included NSCLC cases [8,9,14,15,17,18,21,24,25,28,30,31,32,33,34,35,36,38,39,40,41,42,43,44,45,46,47,48,50,51,53,54,55,56], six studies included only ADC cases [19,23,26,27,29,49], three studies included only SCC cases [10,16,20], and one study included small cell lung cancer (SCLC) cases [57]. Regarding subgroup analyses, five studies performed histology-specific analyses (Table 1 and Table 2) [17,28,34,48,50], and eight studies performed analyses according to cancer stage (Table S2) [26,28,30,39,45,48,50,53]. In histology-specific analyses, several studies reported differential sensitivity, specificity or AUC values in different histological subtypes (ADC and SCC) for the same miRNA or miRNA panel [17,28,34,48,50]. This indicates that miRNAs could be differentially expressed in different histological subtypes of LC, but no histology-specific miRNA could be identified (Table 1 and Table 2). In analyses according to cancer stage, several studies showed that diagnostic efficacy of either miRNAs or miRNA panels in advanced stage of LC seems to be better compared to early stage of LC, however, the differences with respect to AUCs were rather limited (Table S2). 

Of the 22 miRNAs reported at least twice, miR-21 was most frequently reported (13 studies), followed by miR-145, miR-20a, miR-24, miR-223, miR-155, miR-25, miR-152, miR-125a-5p, miR-126, miR-221 and miR-93 (all three to five studies) (Table 3). Of note, higher frequency of reporting of the investigated markers does not go along with higher values of diagnostic performance parameters. For example, the median sensitivity and specificity of miR-21 were 69% (46.3–91%) and 71.9% (60–92%) respectively.

### 3.4. Direction of Dysregulation of Circulating miRNAs

Of the 46 studies, 45 studies described the direction of dysregulation of miRNAs in blood, and only one study did not report information on miRNA dysregulation (Table S1). Among the 22 miRNAs reported in ≥2 studies, inconsistent directions were reported for six miRNAs (Table S3), whereas consistent direction of dysregulation was observed for most of the markers. For example, up-regulation of miR-24, miR-223, miR-155 and miR-221, and down-regulation of miR-126 were consistently reported in all studies reporting on these miRNAs regardless of histological subtype, stage or sample type (Table S1). In addition, miR-21, the most frequently reported miRNA, was also up-regulated in both plasma and serum samples in all included studies, except one study reporting down-regulation of miR-21 in exosome [8].

### 3.5. Comparison of miRNAs Profiles for LC Detection between Eastern Asian and Western Populations

**Figure 3:**
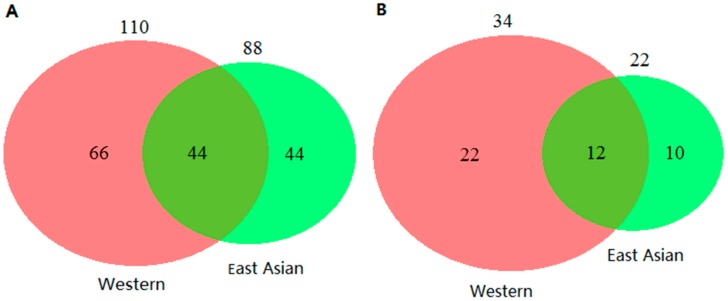
Venn diagrams showing numbers of miRNAs with reported significant associations with lung cancer for Western and East Asian studies. (**A**) miRNAs reported in ≥1 study and (**B**) miRNAs reported in ≥2 studies.

**Figure 4:**
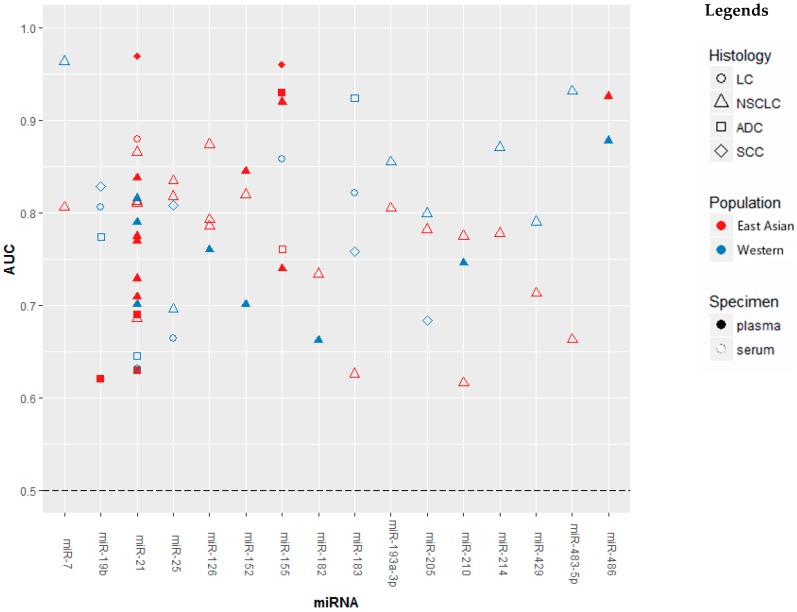
Graphical representation of Western studies versus East Asian studies of the diagnostic performance of miRNAs in lung cancer. Abbreviations: AUC: area under the curve; LC: lung cancer; NSCLC: non-small cell lung cancer; ADC: adenocarcinoma; SCC: squamous cell carcinoma.

We previously performed a systematic review of literature on circulating miRNAs for lung cancer detection in Western populations [12]. A further update of this review until September 17th, 2018 identified a total number of 110 miRNAs, 34 of which were reported in ≥2 studies. We plotted Venn diagrams to illustrate the overlap of identified miRNAs between East Asian and Western populations. Forty-four miRNAs were reported in both populations (Figure 3A), which accounted for 50% of miRNAs reported in the East Asian populations, and 40% of miRNAs reported in the Western populations. Restricting miRNAs reported ≥2 times in each type of population, there were 12 overlapping miRNAs (Figure 3B), which account for 55% of miRNAs reported in East Asian and 35% of miRNAs reported in Western populations. Sixteen miRNAs were evaluated individually in both East Asian and Western populations, AUC values of which are shown in Figure 4. For some miRNAs showing differences in diagnostic performance were observed between East Asian and Western studies, but such differences were also seen within each type of study populations. Detailed information of the 16 miRNAs is provided in Table S4. 

## 4. Discussion

Our systematic literature review identified 46 studies that evaluated a total of 88 miRNA markers for LC diagnosis in East Asian populations. Sixteen of the 46 studies have incorporated individual miRNA markers as panels (with 2–20 markers). Three promising miRNA panels with ≥90% sensitivity and ≥90% specificity were discovered, two of which were verified externally [33,54]. Diagnostic performance of circulating miRNA in East Asian populations was comparable to diagnostic performance in Western populations. Forty-four miRNAs were reported in both populations. No major differences in diagnostic performance by ethnicity of the same miRNA was observed.

In general, the performance of the investigated miRNAs and miRNA panels for detecting LC in East Asian populations appears promising, and in most cases the sum of the sensitivity and specificity by far exceeded 100% (Figure 2A). There were 17 individual miRNAs and 11 miRNA panels for which both sensitivity and specificity above 80% were reported (Figure 2B). Several miRNA panels even showed rather good diagnostic efficiency. For instance, Fan et al. [33] used a panel composed of miR-20a-5p, miR-16-5p and miR-15b-5p in serum to discriminate NSCLC cases from healthy controls, and the sensitivity and specificity reached 94% and 94%, respectively, in the validation set. Chen et al. [54] used a 10-miRNA panel in serum, and reported 93% sensitivity and 90% specificity in the validation set. Several miRNA panels were reported to be useful for the detection of early stage LC, with verified AUC values over 0.85 [8,10,48]. However, most studies evaluated miRNAs or miRNA panels in samples of stage I–IV LC cases, the diagnostic efficiency of the reported markers or panels for early stage LC needs to be validated in true screening settings. Differences in the subgroup analyses for histology and stage of LC were rather small and relevant data were not sufficient to obtain robust results.

Aberrant expression of specific circulating miRNAs may provide important information for distinguishing LC histological subtypes or stages. However, subgroup analyses with respect to LC histological subtypes or stages have only been performed in a small proportion of the included studies [17,26,28,30,34,39,45,48,50,53]. Even though histology-specific analysis identified variations of diagnostic indicators of miRNA in different subtypes, no histology-specific miRNA was discovered due to the overlap in profiles. Consistent findings were also observed in studies conducted in the Western populations. Stage-specific analyses in both the Eastern Asian and Western populations showed that diagnostic efficacy was better in advanced stage compared to early stage; however, the differences with respect to AUC were quite small.

Over the past decade, studies focusing on miRNA and LC have been emerging rapidly and have identified a number of LC-specific miRNA expression profiles. However, these miRNA expression profiles are not consistent among different studies [58,59,60,61]. In this systematic review, the degree of overlap of LC-specific miRNAs reported by various studies was found to be low and inconsistent directions of dysregulation of miRNAs was observed. Similar findings were also noted among the Western studies [12]. To be used as biomarkers for LC screening, miRNAs should show consistent degrees and direction of dysregulation in different settings [62,63]. Possible sources of inconsistency of results across studies could include the use of different biospecimen or different analytical platforms. Ideally, future studies should involve different types of biospecimen and different types of analytical platforms in the same study population in order to further elucidate the role of these factors in miRNA profiling.

Further causes of the heterogeneity of reported miRNA biomarkers may be the differences in the characteristics of study populations. For the included studies in both East Asian and Western populations, we extracted demographic information such as age, sex and country, as these factors may affect the identification of miRNA markers [11,64,65]. Cigarette smoking could also induce the dysregulation of some circulating miRNAs, which might be associated with smoking-related LC [47,66,67]. However, information on smoking status was incomprehensive in most studied populations, especially in controls. Sample sizes of the majority of both East Asian and Western studies were rather small, which might have resulted in substantial random variation of LC-specific miRNAs identified among studies. In addition, miRNA profiles may change in different phases of cancer [68]. Most studies in both East Asian and Western populations however recruited LC cases with various stage proportions.

The use of different miRNA detection protocols could also affect the identification of LC-specific miRNAs. Pre-analytical factors such as speed and duration of centrifugation may affect the amount of cell debris remaining in the sample supernatant (especially in plasma samples) and cause miRNA contaminations [69,70,71,72]. Only a fraction of included studies in both East Asian and Western populations have applied a second high-speed centrifugation step to reduce the remaining cell debris (Table S5). Moreover, reports on most studies did not specifically address potential hemolysis, which could result in an increase of multiple miRNA levels [70,71,73,74]. Analytical factors such as differences in miRNA extraction and quantification methods could also contribute to the variability of identified LC-specific miRNAs [75]. The extraction methods in the included studies were diverse (Table S5), only few studies in both East Asian and Western populations have used miRNeasy kit which has been suggested to have better extraction efficiency compared to other kits [76,77]. In addition, a very important yet unresolved issue is the normalization of miRNA expression data. Circulating miR-16 is usually used as endogenous control for normalization (Table S5), however, it shows an unstable expression in the circulation of cancer patients as well as in hemolytic samples [71,78,79]. Several new bioinformatics tools such as miRNA ratios and differentially expressed miRNA pairs have been developed to build up miRNA panels to reduce the analytical bias [80,81,82]. These attempts so far have shown promising results but still require further validation.

The herein described circulating miRNAs have several advantages over some other markers of cancer detection: (i) miRNAs are extremely stable in cell-free fluids and in less than ideal sample handling conditions [79,83,84], (ii) they can be measured repeatedly over time in a non-invasive manner [85], (iii) they can be used to predict cancer in high-risk populations years in advance [80,82,86] and (iv) the cost of analysis is relative low. Still, other blood-based markers have been proposed for LC detection, such as cytokeratin 19 fragment 21-1 (CYFRA21-1), carcinoembryonic antigen (CEA), tissue polypeptide specific antigen (TPS) or neuron-specific enolase (NSE). However, these markers are primarily utilized for monitoring of disease progression and tend to show suboptimal diagnostic value for LC with sensitivity values usually under 50% [81,87]. Sanfiorenzo et al. [88] developed a signature of 11 plasma miRNAs to discriminate healthy individuals from NSCLC patients which yielded 85% sensitivity and 82.9% specificity. This estimate of sensitivity was compared favorably with an estimated sensitivity of blood-based biomarkers, such as CYFRA 21-1, TPS and CEA, which ranged from 34% to 53% in the same study population. Recently, DNA methylation markers have showed good diagnostic efficacy for LC. Zhang et al. [89] used a combination of F2RL3 methylation in whole blood and smoking exposure to predict LC incidence with an AUC value of 0.86 for participants ≥65 years. Since miRNA markers and DNA methylation markers both shown good diagnostic performance for LC, the combination of the two may offer an improvement of diagnostic efficiency in LC.

## 5. Conclusions

Our systematic review suggests a number of circulating miRNAs to be promising candidates for noninvasive LC detection in East Asian populations. The heterogeneity of reported LC-specific miRNA profiles in published studies needs to be addressed and protocols for the standardization of miRNA analysis procedures need to be put into place. Larger prospective studies, the improvement of miRNA detection technologies, the minimization of pre-analytical or analytical variability as well as the development of new analysis methods will be crucial to further reduce the bias in measurement and analysis and improve the diagnostic performance. Most importantly, however, will be the rigorous independent validation of identified promising miRNA algorithms or their combination with other biomarkers in prospective screening cohorts.

## Figures and Tables

**Table 1:** Diagnostic performance of miRNA markers in lung cancer in East Asian populations.

Study ^a^	Cases vs. Controls			Specimen	Histology	Stage	miRNA	SEN	SPE	AUC	*p*-Value
	Number	Age (y)	Male (%)								
Zhao, 2018 [14] ^b^	108/54	/	61/NA	serum	NSCLC	I–IV	miR-141	/	/	0.86	<0.001 ^c^
Sun, 2018 [15] ^b^	196/77	/	58/NA	serum	NSCLC	I–IV	miR-770	68	89	0.84	<0.01 ^c^
Shan, 2018 [16] ^b^	102/101	/	100/100	plasma	SCC	I–III	miR-181a-5p	/	/	0.73	<0.001 ^c^
							miR-21-5p	/	/	0.74	<0.001 ^c^
							miR-106a-5p	/	/	0.74	<0.001 ^c^
							miR-93-5p	/	/	0.69	<0.001 ^c^
Qin, 2018 [17]	146/40	63/NA	73/NA	serum	NSCLC	I–IV	miR-342-3p	60	94	0.89	<0.01 ^c^
	78/40	/	/		SCC		miR-342-3p	83	76	0.87	<0.01 ^c^
	68/40	/	/		ADC		miR-342-3p	83	82	0.90	<0.01 ^c^
Bao, 2018 [18] ^b^	80/75	/	61/60	serum	NSCLC	I–IV	miR-10a-5p	66	73	0.71	<0.0001 ^c^
							miR-196a-5p	68	78	0.79	0.0018 ^c^
Zhang, 2017 [20] ^b^	102/108	/	100/100	Serum	SCC	I–III	miR-106a-5p	/	/	0.83	<0.001 ^c^
							miR-20a-5p	/	/	0.80	<0.001 ^c^
							miR-93-5p	/	/	0.82	<0.001 ^c^
Wang, 2017 [24]	127/60	58/59	62/58	serum	NSCLC	I–IV	miR-98	80	82	0.86	<0.01 ^c^
Zhou, 2017 [19] ^b^	108/94	/	43/46	plasma	ADC	I–IV	miR-19b-3p	/	/	0.62	<0.001 ^c^
							miR-21-5p	/	/	0.69	<0.001 ^c^
							miR-221-3p	/	/	0.68	<0.001 ^c^
							miR-409-3p	/	/	0.61	<0.001 ^c^
							miR-425-5p	/	/	0.66	<0.001 ^c^
							miR-584-5p	/	/	0.69	<0.001 ^c^
Zhang, 2017 [21]	129/83	60/60	63/58	plasma	NSCLC	I–II	miR-145	81	89	0.89	<0.0001 ^c^
							miR-20a	80	88	0.89	<0.0001 ^c^
							miR-21	78	86	0.84	<0.001 ^c^
							miR-223	70	84	0.81	<0.001 ^c^
Yu, 2017 [22] ^b^	50/30	/	82/83	plasma	SCLC	I–IV	miR-92a-2-5p	56	100	0.76	<0.001 ^c^
Yang, 2017 [23] ^b^	113/30	/	52/NA	serum	ADC	I–IV	miR-31	/	/	0.84	<0.01 ^c^
Shang, 2017 [25]	127/112	55/44	65/56	serum	NSCLC	I–IV	miR-22	99	84	0.92	<0.001
							miR-126	83	96	0.87	<0.001
Lv, 2017 [26]	120/120	60/59	52/51	serum	ADC	I–IV	miR-103	/	/	0.80	<0.0001 ^c^
							miR-146a	/	/	0.90	<0.0001 ^c^
							miR-151	/	/	0.85	<0.0001 ^c^
							miR-221	/	/	0.79	<0.0001 ^c^
							miR-222	/	/	0.86	<0.0001 ^c^
							miR-223	/	/	0.91	<0.0001 ^c^
Zhu, 2016 [9]	112/40	59/58	54/55	serum	NSCLC	0–IIIB	miR-182	63	80	0.73	<0.0001
							miR-183	41	83	0.63	0.0091
							miR-210	34	100	0.62	0.0121
							miR-126	61	93	0.79	<0.0001
Wang, 2016 [27]	82/91	/	/	plasma	ADC	I–II	miR-628-3p	43	91	0.73	<0.001 ^c^
							miR-339-3p	65	71	0.72	<0.001 ^c^
							miR-425-3p	67	68	0.73	<0.001 ^c^
							miR-532	54	80	0.66	<0.001 ^c^
Wang, 2016 [28]	54/15	60/51	/	serum	NSCLC	I–IV	miR-1244	82	80	0.83	/
	26/15	NA/51	/		ADC		miR-1244	77	80	0.79	<0.05 ^c^
	18/15	NA/51	/		SCC		miR-1244	83	80	0.85	<0.05 ^c^
Sun, 2016 [30]	50/60	67/62	76/78	serum	NSCLC	I–IV	miR-21	/	/	0.87	0.000
Su, 2016 [32] ^b^	100/100	/	65/70	plasma	NSCLC	I–III	miR-195	78	86	0.89	<0.01 ^c^
Peng, 2016 [32]	120/71	60/58	72/62	serum	NSCLC	I–IV	miR-1254	**97**	**39**	**0.68**	0.000
							miR-485-5p	**95**	**63**	**0.79**	0.000
							miR-574-5p	**99**	**28**	**0.64**	0.002
Gao, 2016 [10]	90/90	62/62	80/78	plasma	SCC	I	miR-324-3p	**72**	**76**	**0.79**	/
							miR-1285	**87**	**78**	**0.85**	/
Fan, 2016 [33] ^d^	94/58	60/58	54/53	serum	NSCLC	I–IIIB	miR-15b-5p	52	74	/	0.006 ^c^
							miR-16-5p	88	86	/	0.0001 ^c^
							miR-17-5p	82	68	/	0.0001 ^c^
							miR-19-3p	83	67	/	0.0001 ^c^
							miR-20a-5p	76	90	/	0.0001 ^c^
							miR-28-3p	71	79	/	0.0001 ^c^
							miR-92a-3p	87	66	/	0.0001 ^c^
Zhou, 2015 [34]	87/61	59/56	63/52	serum	NSCLC	I–IV	miR-194	/	/	0.66	/
							miR-652	/	/	0.82	/
							miR-660	/	/	0.71	/
	52/61	NA/56	NA/52		ADC		miR-194	/	/	0.63	0.0192 ^c^
							miR-652	/	/	0.82	<0.0001 ^c^
							miR-660	/	/	0.72	<0.0001 ^c^
	35/61	NA/56	NA/52		SCC		miR-194	/	/	0.70	0.0007 ^c^
							miR-652	/	/	0.81	<0.0001 ^c^
							miR-660	/	/	0.70	0.0003 ^c^
Zhao, 2015 [35]	80/60	58/55	61/52	serum	NSCLC	/	miR-21	74	72	0.81	0.001 ^c^
Yang, 2015 [36] ^b^	152/300	/	65/69	serum	NSCLC	I–IV	miR-148a	85	83	0.90	<0.001 ^c^
							miR-148b	83	83	0.90	<0.001 ^c^
							miR-152	75	77	0.82	<0.001 ^c^
							miR-21	69	71	0.81	<0.001 ^c^
Yan, 2015 [37] ^b^	300/300	/	/	plasma	Any LC	I–IV	miR-31	77	75	0.79	<0.001 ^c^
Wang, 2015 [38]	70/70	64/64	61/61	serum	NSCLC	/	miR-125a-5p	74	56	0.71	<0.0001 ^c^
							miR-145	93	61	0.84	<0.0001 ^c^
							miR-146a	84	59	**0.78**	<0.0001 ^c^
Wang, 2015 [39]	94/111	NA/60	NA/52	serum	NSCLC	IA–IIB	miR-125a-5p	88	75	**0.83**	/
							miR-25	83	75	**0.82**	/
							miR-126	83	63	**0.79**	/
Wang, 2015 [40] ^e^	63/63	62/60	78/59	serum	NSCLC	I–IV	miR-483-5p	/	/	**0.66**	0.002
							miR-193a-3p	/	/	**0.81**	<0.0001
							miR-214	/	/	**0.78**	<0.0001
							miR-25	/	/	**0.84**	<0.0001
							miR-7	/	/	**0.81**	<0.0001
Li, 2015 [41]	11/11	59/55	64/55	plasma	NSCLC	I–IIIA	miR-486	91	82	0.93	0.0008 ^c^
							miR-150	82	82	0.75	0.0488 ^c^
Guo, 2015 [42] ^b^	126/50	/	64/NA	plasma	NSCLC	I–IV	miR-204	**76**	**82**	**0.81**	<0.001 ^c^
Dou, 2015 [43]	120/360	63/NA	60/NA	plasma	NSCLC	I–IV	let-7c	72	78	0.71	0.006
							miR-152	86	81	0.85	0.0002
Zhu, 2014 [44]	36/44	/	/	serum	NSCLC	I	miR-125a-5p	54	75	0.65	0.021
							let-7e	50	83	0.64	0.0317
Zhu, 2014 [45]	70/48	59/NA	80/NA	serum	NSCLC	I–IV	miR-29c	66	74	0.68	0.0004
							miR-429	54	81	0.71	<0.0001
Li, 2014 [46] ^b^	514/54	/	53/NA	serum	NSCLC	I–IV	miR-499	74	93	0.91	<0.001 ^c^
Huang, 2014 [47]	53/65	57/55	/	serum	NSCLC	/	let-7i-3p	/	/	**0.89**	<0.001 ^c^
							miR-154-5p	/	/	**0.96**	<0.001 ^c^
Geng, 2014 [48] ^b^	126/60	/	69/60	plasma	NSCLC	I–II	miR-20a	**83**	**81**	**0.89**	<0.001 ^c^
							miR-223	**87**	**86**	**0.94**	<0.001 ^c^
							miR-21	**67**	**68**	**0.77**	<0.001 ^c^
							miR-155	**86**	**84**	**0.92**	<0.001 ^c^
							miR-145	**70**	**68**	**0.77**	<0.001 ^c^
	45/60	/	/		ADC	I–II	miR-20a	**87**	**83**	**0.90**	/
							miR-223	**80**	**82**	**0.91**	/
							miR-21	**60**	**60**	**0.63**	/
							miR-155	**87**	**87**	**0.93**	/
							miR-145	**71**	**70**	**0.77**	/
	64/60	/	/		SCC	I–II	miR-20a	**97**	**97**	**0.98**	/
							miR-223	**95**	**93**	**0.98**	/
							miR-21	**91**	**90**	**0.97**	/
							miR-155	**92**	**90**	**0.96**	/
							miR-145	**95**	**95**	**0.97**	/
Gao, 2014 [49]	36/32	55/53	31/31	serum	ADC	I–IV	miR-155	72	69	0.76	0.000
Tang, 2013 [50]	34/32	65/66	65/81	plasma	NSCLC	I–III	miR-21	**53**	**72**	**0.71**	/
							miR-145	**56**	**56**	**0.66**	/
							miR-155	**68**	**66**	**0.74**	/
Li, 2013 [51]	60/30	54/57	70/50	serum	NSCLC	I–IV	miR-210	79	74	0.78	<0.005 ^c^
Wang, 2012 [52]	31/39	61/46	71/23	serum	Any LC	I–IV	miR-21	87	74	0.88	<0.001 ^c^
Le, 2012 [53]	82/50	59/NA	56/NA	serum	NSCLC	I–IV	miR-21	46	92	0.69	0.0058 ^c^
							miR-205	85	72	0.78	0.0298 ^c^
							miR-30d	76	80	0.74	0.0147 ^c^
							miR-24	76	64	0.83	<0.0001 ^c^
Wei, 2011 [55]	77/36	60/56	71/75	plasma	NSCLC	I–IV	miR-21	61	83	0.73	<0.0001
Wei, 2011 [56]	63/30	61/57	71/67	plasma	NSCLC	I–IV	miR-21	76	70	0.78	<0.0001

^a^ all studies listed in the table were from China; ^b^ no mean age or median age but age distribution reported; ^c^ *p*-value represents the difference of miRNA levels between cases and controls (all other *p*-values represent the statistical significance of AUC values); ^d^ miRNAs detected with fluorescence quantum dots liquid bead array (all other studies detected with qRT-PCR); ^e^ USA validation set not included; SENs, SPEs and AUCs in bold fonts represent results from validation set (non-bold fonts represent results without validation). Abbreviations: SEN: sensitivity; SPE: specificity; AUC: area under the curve; LC: lung cancer; NSCLC: non-small cell lung cancer; ADC: adenocarcinoma; SCC: squamous cell carcinoma; NA: not available.

**Table 2:** Diagnostic performance of miRNA panels in lung cancer in East Asian populations.

Study ^a^	Cases vs. Controls			Specimen	Histology	Stage	miRNA	SEN	SPE	AUC
	Number	Age (y)	Male (%)							
Shan, 2018 [16] ^b^	15/15	/	100/100	plasma	SCC	I–III	Panel A	/	/	**0.91**
Zhang, 2017 [20] ^b^	34/36	/	100/100	serum	SCC	I–III	Panel B	/	/	**0.95**
Zhou, 2017 [19] ^b^	33/30	/	45/47	plasma	ADC	I–IV	Panel C	**73**	**80**	**0.84**
Zhang, 2017 [21]	129/83	60/60	63/58	plasma	NSCLC	I–II	Panel D	82	90	0.90
LV, 2017 [26]	120/120	60/59	52/51	serum	ADC	I–IV	Panel E	**84**	**91**	**0.95**
	72/120	NA/59	NA/51			I		/	/	**0.94**
	31/120	NA/59	NA/51			II		/	/	**0.97**
	10/120	NA/59	NA/51			III		/	/	**0.95**
Jin, 2017 [8]	47/13	/	/	exosome	NSCLC	I	Panel F	**80**	**92**	**0.90**
Wang, 2016 [27]	82/91	/	/	plasma	ADC	I–II	Panel G	90	99	0.98
							Panel H	92	98	0.97
Tai, 2016 [29]	110/52	65/66	56/58	serum	ADC	I–III	Panel I	**89**	**100**	**0.98**
Gao, 2016 [10]	90/90	62/62	80/78	plasma	SCC	I	-324-3p, -1285	**85**	**82**	**0.89**
Fan, 2016 [33] ^c^	70/54	60/58	60/54	serum	NSCLC	I–IIIB	Panel J	**94**	**94**	/
Zhou, 2015 [34]	87/61	59/56	63/52	serum	NSCLC	I–IV	-652, -660	/	/	**0.86**
	52/61	NA/56	NA/52		ADC		-652, -660	/	/	**0.85**
	35/61	NA/56	NA/52		SCC		-652, -660	/	/	**0.87**
Yang, 2015 [36] ^b^	152/300	/	65/69	serum	NSCLC	I–IV	Panel K	96	91	0.98
Wang, 2015 [39]	142/111	61/60	61/52	serum	NSCLC	IA–IV	Panel L	**88**	**83**	**0.93**
Wang, 2015 [40] ^d^	63/63	62/60	78/59	serum	NSCLC	I–IV	Panel M	**89**	**68**	**0.82**
Tang, 2013 [50]	34/32	65/66	65/81	plasma	NSCLC	I–III	-21, -145	**74**	**81**	**0.85**
							-21, -145	**56**	**66**	**0.73**
							-145, -155	**79**	**78**	**0.83**
							Panel N	**77**	**81**	**0.87**
	40/60	NA/66	NA/77		ADC	I–III	Panel N	71	78	/
	9/60	NA/66	NA/77		SCC	I–III	Panel N	67	78	/
Chen, 2012 [54]	200/110	60/59	79/72	serum	NSCLC	I–IV	Panel O	**93**	**90**	**0.97**

^a^ All studies listed in the table were from China, except Tai [29] which was from Japan; ^b^ no mean age or median age but age distribution reported; ^c^ miRNAs detected with fluorescence quantum dots liquid bead array (all other studies detected with qRT-PCR); ^d^ USA validation set not included; SENs, SPEs and AUCs in bold fonts represent results from validation set (non-bold fonts represent results without validation). Panel A: -106a-5p, -20a-5p, -93-5p; Panel B: -181a-5p, -21-5p, -106a-5p, -93-5p; Panel C: -19b-3p, -21-5p, -221-3p, -409-3p, -425-5p, -584-5p; Panel D: -145, -20a, -21, -223; Panel E: -146a, -222, -223; Panel F: let-7b-5p, let-7e-5p, -24-5p, -21-5p; Panel G: -628-3p, -339-3p, -425-3p, -532; Panel H: -628-3p, -425-3p, -532; Panel I (20 miRs): -451, -1290. -636, -30c, -22-3p, -19b, -486-5p, -20b, -93, -34b, -185, -126-5p, -93-3p, -1274a, -142-5p, -628-5p, -486-3p, -425, -645, -24; Panel J: -20a-5p, -16-5p, -15b-5p; Panel K: -148a, -148b, -152, -21; Panel L: -25, -125a-5p, -126; Panel M: -214, -483-5p, -193a-3p, -25, -7; Panel N: -21, -145, -155; Panel O (10 miRs): -20a, -24, -25, -145, -152, -199a-5p, -221, -222, -223, -320. Abbreviations: SEN: sensitivity; SPE: specificity; AUC: area under the curve; LC: lung cancer; NSCLC: non-small cell lung cancer; ADC: adenocarcinoma; SCC: squamous cell carcinoma; NA: not available.

**Table 3:** Summary of studies reporting associations of miRNAs with lung cancer in East Asian populations (only miRNAs that have been reported in ≥2 studies are shown).

Study	miR-21	miR-145	miR-20a	miR-24	miR-223	miR-155	miR-25	miR-152	miR-125a	miR-126	miR-221	miR-93	miR-210	miR-486	miR-425	miR-19b	miR-22	let-7e	miR-146a	miR-222	miR-31	miR-106a
Shan, 2018 [16]	○↑											○↑										○↑
Zhang, 2017 [20]			○↑									○↑										○↑
Zhou, 2017 [19]	○↑										○↑					○↑						
Zhang, 2017 [21]	○↑	○↑	○↑		○↑																	
Yu, 2017 [22]																						
Yang, 2017 [23]																					△↑	
Shang, 2017 [25]										△↓							△↑					
Lv, 2017 [26]					○↑						△↑								○↑	○↑		
Jin, 2017 [8]	○↓			○↑														○↓				
Zhu, 2016 [9]										△↓			△↑									
Wang, 2016 [27]															○↑							
Tai, 2016 [29]				○								○		○	○	○	○					
Sun, 2016 [30]	△↑																					
Fan, 2016 [33]			○↓																			
Zhao, 2015 [35]	△↑																					
Yang, 2015 [36]	○↑							○↓														
Yan, 2015 [37]																					△↑	
Wang, 2015 [38]		△↑							△↑										△↑			
Wang, 2015 [39]							○↓		○↓	○↓												
Wang, 2015 [40]							○↑															
Li, 2015 [41]														△↑								
Dou, 2015 [43]								△↓														
Zhu, 2014 [44]									△↓									△↓				
Geng, 2014 [48]	△↑	△↑	△↑		△↑	△↑																
Gao, 2014 [49]						△↑																
Tang, 2013 [50]	○↑	○↓				○↑																
Li, 2013 [51]													△↑									
Wang, 2012 [52]	△↑																					
Le, 2012 [53]	△↑			△↑																		
Chen, 2012 [54]		○↑	○↑	○↑	○↑		○↑	○↑			○↑									○↑		
Wei, 2011 [55]	△↑																					
Wei, 2011 [56]	△↑																					
Total	13	5	5	4	4	3	3	3	3	3	3	3	2	2	2	2	2	2	2	2	2	2

○ represents miRNAs which are part of a panel; △ represents miRNAs which have only been analyzed individually and not as a part of a miRNA panel; ↑ represents up-regulation; ↓ represents down-regulation; Table S1 provides all miRNAs for which significant associations with lung cancer have been reported.

## Supplementary Materials

The following are available online at https://www.mdpi.com/2072-6694/11/3/415/s1, Figure S1: Risk of bias and applicability concerns graph: review authors’ judgements about each domain presented as percentages across included studies, Figure S2: Risk of bias and applicability concerns summary: review authors’ judgements about each domain for each included study, Table S1: Summary of studies reporting significant associations of miRNAs with lung cancer in East Asian populations, Table S2: Diagnostic performance of miRNAs and miRNA panels according to lung cancer stage in East Asian populations, Table S3: MiRNAs for which opposite directions of dysregulation were reported in lung cancer blood samples, Table S4: East Asian studies versus Western studies of miRNAs for lung cancer detection, Table S5: Protocols of blood miRNA detection.
