# Supplementary material for: Circulating MicroRNA Biomarkers for Lung Cancer Detection in East Asian Populations
Source: Cancers (Basel). 2019 Mar 23;11(3):415. doi: 10.3390/cancers11030415 (PMC6468694; doi:10.3390/cancers11030415)
Supplement: Supplementary file 1 [file cancers-11-00415-s001.zip › cancers-462334-suppl-layout.docx]

Supplementary Materials: Circulating MicroRNA Biomarkers for Lung Cancer Detection in East Asian Populations

Haixin Yu, Zhong Guan, Katarina Cuk, Yan Zhang and Hermann Brenner

**Table S1:** Summary of studies reporting significant associations of miRNAs with lung cancer in East Asian populations.

**Table S2.** Diagnostic performance of miRNAs and miRNA panels according to lung cancer stage in East Asian populations.

**Table S3.** MiRNAs for which opposite directions of dysregulation were reported in lung cancer blood samples.

**Table S4.** East Asian studies versus Western studies of miRNAs for lung cancer detection.

**Table S5.** Protocols of blood miRNA detection.

The tables were all in the excel.


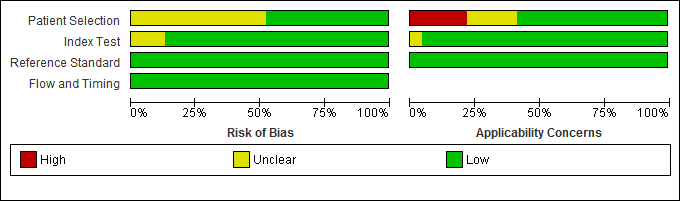


**Figure 1.** Risk of bias and applicability concerns graph: review authors' judgements about each domain presented as percentages across included studies.


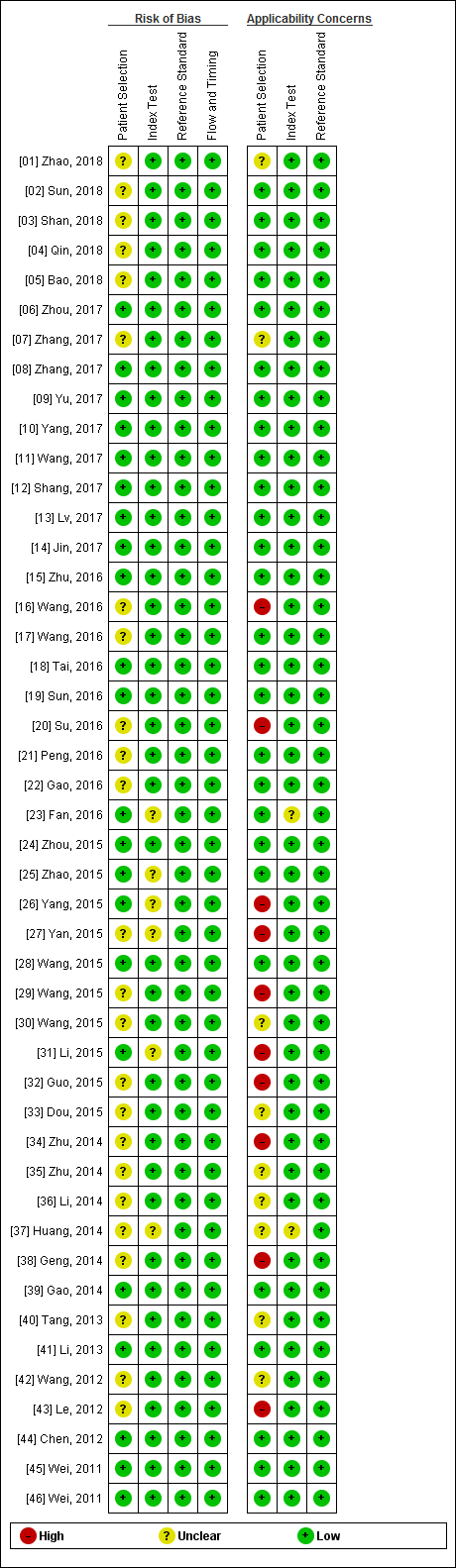


**Figure S2.** Risk of bias and applicability concerns summary: review authors' judgements about each domain for each included study.

East Asian studies [1–46]

Western studies [30,47–64]

References

1. Zhao, Y.Z. The diagnostic and prognostic role of circulating miR-141 expression in non-small-cell lung cancer patients. *Int. J. Clin Exp. Patho.* **2018**, *11*, 2597–2604.

2. Sun, B.; Liu, H.F.; Ding, Y.; Li, Z. Evaluating the diagnostic and prognostic value of serum miR-770 in non-small cell lung cancer. *Eur. Rev. Med. Pharmacol. Sci.* **2018**, *22*, 3061–3066.

3. Shan, X.; Zhang, H.; Zhang, L.; Zhou, X.; Wang, T.S.; Zhang, J.Y.; Shu, Y.Q.; Zhu, W.; Wen, W.; Liu, P. Identification of four plasma microRNAs as potential biomarkers in the diagnosis of male lung squamous cell carcinoma patients in China. *Cancer Med.* **2018**, *7*, 2370–2381, doi:10.1002/cam4.1490.

4. Qin, Y.Z.; Zhou, X.Y.; Huang, C.; Li, L.; Liu, H.S.; Liang, N.X.; Chen, Y.Y.; Ma, D.J.; Han, Z.J.; Xu, X.H.; et al. Serum miR-342-3p is a novel diagnostic and prognostic biomarker for non-small cell lung cancer. *Int. J. Clin. Exp. Patho.* **2018**, *11*, 2742–2748.

5. Bao, M.; Pan, S.; Yang, W.L.; Chen, S.; Shan, Y.B.; Shi, H.C. Serum miR-10a-5p and miR-196a-5p as non-invasive biomarkers in non-small cell lung cancer. *Int. J. Clin. Exp. Patho.* **2018**, *11*, 773–780.

6. Zhou, X.; Wen, W.; Shan, X.; Zhu, W.; Xu, J.; Guo, R.H.; Cheng, W.F.; Wang, F.; Qi, L.W.; Chen, Y.; et al. A six-microRNA panel in plasma was identified as a potential biomarker for lung adenocarcinoma diagnosis. *Oncotarget* **2017**, *8*, 6513–6525, doi:10.18632/oncotarget.14311.

7. Zhang, L.; Shan, X.; Wang, J.; Zhu, J.; Huang, Z.; Zhang, H.; Zhou, X.; Cheng, W.F.; Shu, Y.Q.; Zhu, W.; et al. A three-microRNA signature for lung squamous cell carcinoma diagnosis in Chinese male patients. *Oncotarget* **2017**, *8*, 86897–86907, doi:10.18632/oncotarget.19666.

8. Zhang, H.; Mao, F.; Shen, T.; Luo, Q.; Ding, Z.; Qian, L.; Huang, J. Plasma miR-145, miR-20a, miR-21 and miR-223 as novel biomarkers for screening early-stage non-small cell lung cancer. *Oncol. Lett.* **2017**, *13*, 669–676, doi:10.3892/ol.2016.5462.

9. Yu, Y.L.; Zuo, J.C.; Tan, Q.; Thin, K.Z.; Li, P.; Zhu, M.; Yu, M.X.; Fu, Z.M.; Liang, C.Z.; Tu, J.C. Plasma miR-92a-2 as a biomarker for small cell lung cancer. *Cancer Biomark.* **2017**, *18*, 319–327, doi:10.3233/cbm-160254.

10. Yang, M.; Xiao, L.B.; Zhang, Y.H.; Li, G.J.; Zhou, J.W. Clinical significance of serum miR-31 as a predictive biomarker for lung adenocarcinoma. *Int. J. Clin. Exp. Patho.* **2017**, *10*, 4668–4674.

11. Wang, K.C.; Dong, L.Y.; Fang, Q.M.; Xia, H.W.; Hou, X.L. Low serum miR-98 as an unfavorable prognostic biomarker in patients with non-small cell lung cancer. *Cancer Biomark.* **2017**, *20*, 283–288, doi:10.3233/cbm-170124.

12. Shang, A.Q.; Xie, Y.N.; Wang, J.; Sun, L.; Wei, J.; Lu, W.Y.; Lan, J.Y.; Wang, W.W.; Wang, L.; Wang, L.L. Predicative values of serum microRNA-22 and microRNA-126 levels for non-small cell lung cancer development and metastasis: A case-control study. *Neoplasma* **2017**, *64*, 453–459, doi:10.4149/neo_2017_317.

13. Lv, S.G.; Xue, J.; Wu, C.Y.; Wang, L.; Wu, J.; Xu, S.J.; Liang, X.H.; Lou, J.T. Identification of A Panel of Serum microRNAs as Biomarkers for Early Detection of Lung Adenocarcinoma. *J. Cancer* **2017**, *8*, 48–56, doi:10.7150/jca.16644.

14. Jin, X.C.; Chen, Y.F.; Chen, H.B.; Fei, S.R.; Chen, D.D.; Cai, X.N.; Liu, L.; Lin, B.C.; Su, H.F.; Zhao, L.H.; et al. Evaluation of Tumor-Derived Exosomal miRNA as Potential Diagnostic Biomarkers for Early-Stage Non-Small Cell Lung Cancer Using Next-Generation Sequencing. *Clin. Cancer Res.* **2017**, *23*, 5311–5319, doi:10.1158/1078-0432.ccr-17-0577.

15. Zhu, W.Y.; Zhou, K.Y.; Zha, Y.; Chen, D.D.; He, J.Y.; Ma, H.J.; Liu, X.G.; Le, H.B.; Zhang, Y.K. Diagnostic Value of Serum miR-182, miR-183, miR-210, and miR-126 Levels in Patients with Early-Stage Non-Small Cell Lung Cancer. *PLoS ONE* **2016**, *11*, doi:10.1371/journal.pone.0153046.

16. Wang, Y.; Zhao, H.; Gao, X.J.; Wei, F.; Zhang, X.W.; Su, Y.J.; Wang, C.L.; Li, H.; Ren, X.B. Identification of a three-miRNA signature as a blood-borne diagnostic marker for early diagnosis of lung adenocarcinoma. *Oncotarget* **2016**, *7*, 26070–26086, doi:10.18632/oncotarget.8429.

17. Wang, W.Z.; Li, W.L.; Ding, M.J.; Yuan, H.N.; Yang, J.; Meng, W.; Jin, E.; Wang, X.J.; Ma, S.L. Identification of miRNAs as non-invasive biomarkers for early diagnosis of lung cancers. *Tumor Biol.* **2016**, *37*, 16287–16293, doi:10.1007/s13277-016-5442-y.

18. Tai, M.C.; Yanagisawa, K.; Nakatochi, M.; Hotta, N.; Hosono, Y.; Kawaguchi, K.; Naito, M.; Taniguchi, H.; Wakai, K.; Yokoi, K.; et al. Blood-borne miRNA profile-based diagnostic classifier for lung adenocarcinoma. *Sci. Rep.* **2016**, *6*, doi:10.1038/srep31389.

19. Sun, M.Z.; Song, J.X.; Zhou, Z.W.; Zhu, R.; Jin, H.; Ji, Y.Q.; Lu, Q.; Ju, H.X. Comparison of Serum MicroRNA21 and Tumor Markers in Diagnosis of Early Non-Small Cell Lung Cancer. *Dis. Markers* **2016**, 10.1155/2016/3823121, doi:10.1155/2016/3823121.

20. Su, K.L.; Zhang, T.C.; Wang, Y.R.; Hao, G.J. Diagnostic and prognostic value of plasma microRNA-195 in patients with non-small cell lung cancer. *World J. Surg. Oncol.* **2016**, *14*, doi:10.1186/s12957-016-0980-8.

21. Peng, H.; Wang, J.; Li, J.; Zhao, M.; Huang, S.K.; Gu, Y.Y.; Li, Y.; Sun, X.J.; Yang, L.; Luo, Q.; et al. A circulating non-coding RNA panel as an early detection predictor of non-small cell lung cancer. *Life Sci.* **2016**, *151*, 235–242, doi:10.1016/j.lfs.2016.03.002.

22. Gao, X.J.; Wang, Y.; Zhao, H.; Wei, F.; Zhang, X.W.; Su, Y.J.; Wang, C.L.; Li, H.; Ren, X.B. Plasma miR-324-3p and miR-1285 as diagnostic and prognostic biomarkers for early stage lung squamous cell carcinoma. *Oncotarget* **2016**, *7*, 59664–59675, doi:10.18632/oncotarget.11198.

23. Fan, L.H.; Qi, H.W.; Teng, J.L.; Su, B.; Chen, H.; Wang, C.H.; Xia, Q. Identification of serum miRNAs by nano-quantum dots microarray as diagnostic biomarkers for early detection of non-small cell lung cancer. *Tumor Biol.* **2016**, *37*, 7777–7784, doi:10.1007/s13277-015-4608-3.

24. Zhou, C.C.; Chen, Z.L.; Dong, J.S.; Li, J.G.; Shi, X.J.; Sun, N.; Luo, M.; Zhou, F.; Tan, F.W.; He, J. Combination of serum miRNAs with Cyfra21-1 for the diagnosis of non-small cell lung cancer. *Cancer Lett.* **2015**, *367*, 138–146, doi:10.1016/j.canlet.2015.07.015.

25. Zhao, W.; Zhao, J.J.; Zhang, L.; Xu, Q.F.; Zhao, Y.M.; Shi, X.Y.; Xu, A.G. Serum miR-21 level: A potential diagnostic and prognostic biomarker for non-small cell lung cancer. *Int. J. Clin. Exp. Med.* **2015**, *8*, 14759–14763.

26. Yang, J.S.; Li, B.J.; Lu, H.W.; Chen, Y.; Lu, C.; Zhu, R.X.; Liu, S.H.; Yi, Q.T.; Li, J.; Song, C.H. Serum miR-152, miR-148a, miR-148b, and miR-21 as novel biomarkers in non-small cell lung cancer screening. *Tumor Biol.* **2015**, *36*, 3035–3042, doi:10.1007/s13277-014-2938-1.

27. Yan, H.J.; Ma, J.Y.; Wang, L.; Gu, W. Expression and Significance of Circulating MicroRNA-31 in Lung Cancer Patients. *Med. Sci. Mon.* **2015**, *21*, 722–726, doi:10.12659/msm.893213.

28. Wang, R.J.; Zheng, Y.H.; Wang, P.; Zhang, J.Z. Serum miR-125a-5p, miR-145 and miR-146a as diagnostic biomarkers in non-small cell lung cancer. *Int. J. Clin. Exp. Patho.* **2015**, *8*, 765–771.

29. Wang, P.; Yang, D.W.; Zhang, H.L.; Wei, X.Y.; Ma, T.L.; Cheng, Z.L.; Hong, Q.Y.; Hu, J.; Zhuo, H.J.; Song, Y.L.; et al. Early Detection of Lung Cancer in Serum by a Panel of MicroRNA Biomarkers. *Clin. Lung Cancer* **2015**, *16*, 313, doi:10.1016/j.cllc.2014.12.006.

30. Wang, C.; Ding, M.; Xia, M.D.; Chen, S.D.; Le, A.V.; Soto-Gil, R.; Shen, Y.; Wang, N.; Wang, J.J.; Gu, W.J.; et al. A Five-miRNA Panel Identified From a Multicentric Case-control Study Serves as a Novel Diagnostic Tool for Ethnically Diverse Non-small-cell Lung Cancer Patients. *Ebiomedicine* **2015**, *2*, 1377–1385, doi:10.1016/j.ebiom.2015.07.034.

31. Li, W.S.; Wang, Y.; Zhang, Q.; Tang, L.L.; Liu, X.P.; Dai, Y.H.; Xiao, L.; Huang, S.G.; Chen, L.; Guo, Z.M.; et al. MicroRNA-486 as a Biomarker for Early Diagnosis and Recurrence of Non-Small Cell Lung Cancer. *PLoS ONE* **2015**, *10*, doi:10.1371/journal.pone.0134220.

32. Guo, W.G.; Zhang, Y.X.; Zhang, Y.; Shi, Y.; Xi, J.J.; Fan, H.; Xu, S.T. Decreased expression of miR-204 in plasma is associated with a poor prognosis in patients with non-small cell lung cancer. *Int. J. Mol. Med.* **2015**, *36*, 1720–1726, doi:10.3892/ijmm.2015.2388.

33. Dou, H.L.; Wang, Y.; Su, G.; Zhao, S. Decreased plasma let-7c and miR-152 as noninvasive biomarker for non-small-cell lung cancer. *Int. J. Clin. Exp. Med.* **2015**, *8*, 9291–9298.

34. Zhu, W.Y.; Luo, B.; An, J.Y.; He, J.Y.; Chen, D.D.; Xu, L.Y.; Huang, Y.Y.; Liu, X.G.; Le, H.B.; Zhang, Y.K. Differential Expression of miR-125a-5p and let-7e Predicts the Progression and Prognosis of Non-Small Cell Lung Cancer. *Cancer Invest.* **2014**, *32*, 394–401, doi:10.3109/07357907.2014.922569.

35. Zhu, W.Y.; He, J.Y.; Chen, D.D.; Zhang, B.J.; Xu, L.Y.; Ma, H.J.; Liu, X.G.; Zhang, Y.K.; Le, H.B. Expression of miR-29c, miR-93, and miR-429 as Potential Biomarkers for Detection of Early Stage Non-Small Lung Cancer. *PLoS ONE* **2014**, *9*, doi:10.1371/journal.pone.0087780.

36. Li, M.; Zhang, Q.; Wu, L.; Jia, C.Y.; Shi, F.H.; Li, S.C.; Peng, A.M.; Zhang, G.L.; Song, X.L.; Wang, C.H. Serum miR-499 as a novel diagnostic and prognostic biomarker in non-small cell lung cancer. *Oncol. Rep.* **2014**, *31*, 1961–1967, doi:10.3892/or.2014.3029.

37. Huang, J.K.; Wu, J.J.; Li, Y.Q.; Li, X.; Yang, T.; Yang, Q.Y.; Jiang, Y.G. Deregulation of Serum MicroRNA Expression Is Associated with Cigarette Smoking and Lung Cancer. *Biomed. Res. Int.* **2014**, 10.1155/2014/364316, doi:10.1155/2014/364316.

38. Geng, Q.; Fan, T.; Zhang, B.Y.; Wang, W.; Xu, Y.; Hu, H. Five microRNAs in plasma as novel biomarkers for screening of early-stage non-small cell lung cancer. *Respir. Res.* **2014**, *15*, doi:10.1186/s12931-014-0149-3.

39. Gao, F.; Chang, J.X.; Wang, H.Q.; Zhang, G.J. Potential diagnostic value of miR-155 in serum from lung adenocarcinoma patients. *Oncol. Rep.* **2014**, *31*, 351–357, doi:10.3892/or.2013.2830.

40. Tang, D.F.; Shen, Y.; Wang, M.Z.; Yang, R.H.; Wang, Z.Z.; Sui, A.H.; Jiao, W.J.; Wang, Y.J. Identification of plasma microRNAs as novel noninvasive biomarkers for early detection of lung cancer. *Eur. J. Cancer Prev.* **2013**, *22*, 540–548, doi:10.1097/CEJ.0b013e32835f3be9.

41. Li, Z.H.; Zhang, H.; Yang, Z.G.; Wen, G.Q.; Cui, Y.B.; Shao, G.G. Prognostic significance of serum microRNA-210 levels in nonsmall-cell lung cancer. *J. Int. Med. Res.* **2013**, *41*, 1437–1444, doi:10.1177/0300060513497560.

42. Wang, B.; Zhang, Q.Y. The expression and clinical significance of circulating microRNA-21 in serum of five solid tumors. *J. Cancer Res. Clin. Oncol.* **2012**, *138*, 1659–1666, doi:10.1007/s00432-012-1244-9.

43. Le, H.B.; Zhu, W.Y.; Chen, D.D.; He, J.Y.; Huang, Y.Y.; Liu, X.G.; Zhang, Y.K. Evaluation of dynamic change of serum miR-21 and miR-24 in pre- and post-operative lung carcinoma patients. *Med. Oncol.* **2012**, *29*, 3190–3197, doi:10.1007/s12032-012-0303-z.

44. Chen, X.; Hu, Z.B.; Wang, W.J.; Ba, Y.; Ma, L.J.; Zhang, C.N.; Wang, C.; Ren, Z.J.; Zhao, Y.; Wu, S.J.; et al. Identification of ten serum microRNAs from a genome-wide serum microRNA expression profile as novel noninvasive biomarkers for nonsmall cell lung cancer diagnosis. *Int. J. Cancer* **2012**, *130*, 1620–1628, doi:10.1002/ijc.26177.

45. Wei, J.; Liu, L.K.; Gao, W.; Zhu, C.J.; Liu, Y.Q.; Cheng, T.; Shu, Y.Q. Reduction of plasma MicroRNA-21 is associated with chemotherapeutic response in patients with non-small cell lung cancer. *Chin. J. Cancer Res.* **2011**, *23*, 123–128, doi:10.1007/s11670-011-0123-2.

46. Wei, J.; Gao, W.; Zhu, C.J.; Liu, Y.Q.; Mei, Z.; Cheng, T.; Shu, Y.Q. Identification of plasma microRNA-21 as a biomarker for early detection and chemosensitivity of non-small cell lung cancer. *Chin. J. Cancer* **2011**, *30*, 407–414.

47. Dou, Y.H.; Zhu, Y.; Ai, J.M.; Chen, H.K.; Liu, H.L.; Borgia, J.A.; Li, X.; Yang, F.; Jiang, B.; Wang, J.; et al. Plasma small ncRNA pair panels as novel biomarkers for early-stage lung adenocarcinoma screening. *BMC Genet.* **2018**, *19*, doi:10.1186/s12864-018-4862-z.

48. Powrozek, T.; Kuznar-Kaminska, B.; Dziedzic, M.; Mlak, R.; Batura-Gabryel, H.; Sagan, D.; Krawczyk, P.; Milanowski, J.; Malecka-Massalska, T. The diagnostic role of plasma circulating precursors of miRNA-944 and miRNA-3662 for non-small cell lung cancer detection. *Pathol. Res. Pract.* **2017**, *213*, 1384–1387, doi:10.1016/j.prp.2017.09.011.

49. Zaporozhchenko, I.A.; Morozkin, E.S.; Skvortsova, T.E.; Ponomaryova, A.A.; Rykova, E.Y.; Cherdyntseva, N.V.; Polovnikov, E.S.; Pashkovskaya, O.A.; Pokushalov, E.A.; Vlassov, V.V.; et al. Plasma miR-19b and miR-183 as Potential Biomarkers of Lung Cancer. *PLoS ONE* **2016**, *11*, doi:10.1371/journal.pone.0165261.

50. Powrozek, T.; Krawczyk, P.; Kowalski, D.M.; Kuznar-Kaminska, B.; Winiarczyk, K.; Olszyna-Serementa, M.; Batura-Gabryel, H.; Milanowski, J. Application of plasma circulating microRNA-448, 506, 4316, and 4478 analysis for non-invasive diagnosis of lung cancer. *Tumor Biol.* **2016**, *37*, 2049–2055, doi:10.1007/s13277-015-3971-4.

51. Halvorsen, A.R.; Bjaanaes, M.; LeBlanc, M.; Holm, A.M.; Bolstad, N.; Rubio, L.; Penalver, J.C.; Cervera, J.; Mojarrieta, J.C.; Lopez-Guerrero, J.A.; et al. A unique set of 6 circulating microRNAs for early detection of non-small cell lung cancer. *Oncotarget* **2016**, *7*, 37250–37259, doi:10.18632/oncotarget.9363.

52. Chen, H.K.; Liu, H.L.; Zou, H.Q.; Chen, R.; Dou, Y.H.; Sheng, S.L.; Dai, S.M.; Ai, J.M.; Melson, J.; Kittles, R.A.; et al. Evaluation of Plasma miR-21 and miR-152 as Diagnostic Biomarkers for Common Types of Human Cancers. *J. Cancer* **2016**, *7*, 490–499, doi:10.7150/jca.12351.

53. Wozniak, M.B.; Scelo, G.; Muller, D.C.; Mukeria, A.; Zaridze, D.; Brennan, P. Circulating MicroRNAs as Non-Invasive Biomarkers for Early Detection of Non-Small-Cell Lung Cancer. *PLoS ONE* **2015**, *10*, doi:10.1371/journal.pone.0125026.

54. Powrozek, T.; Krawczyk, P.; Kowalski, D.M.; Winiarczyk, K.; Olszyna-Serementa, M.; Milanowski, J. Plasma circulating microRNA-944 and microRNA-3662 as potential histologic type-specific early lung cancer biomarkers. *Transl. Res.* **2015**, *166*, 315–323, doi:10.1016/j.trsl.2015.05.009.

55. Sozzi, G.; Boeri, M.; Rossi, M.; Verri, C.; Suatoni, P.; Bravi, F.; Roz, L.; Conte, D.; Grassi, M.; Sverzellati, N.; et al. Clinical Utility of a Plasma-Based miRNA Signature Classifier Within Computed Tomography Lung Cancer Screening: A Correlative MILD Trial Study. *J. Clin. Oncol.* **2014**, *32*, 768, doi:10.1200/jco.2013.50.4357.

56. Sanfiorenzo, C.; Ilie, M.I.; Belaid, A.; Barlesi, F.; Mouroux, J.; Marquette, C.H.; Brest, P.; Hofman, P. Two Panels of Plasma MicroRNAs as Non-Invasive Biomarkers for Prediction of Recurrence in Resectable NSCLC. *PLoS ONE* **2013**, *8*, doi:10.1371/journal.pone.0054596.

57. Rani, S.; Gately, K.; Crown, J.; O'Byrne, K.; O'Driscoll, L. Global analysis of serum microRNAs as potential biomarkers for lung adenocarcinoma. *Cancer Biol. Ther.* **2013**, *14*, 1104–1112, doi:10.4161/cbt.26370.

58. Ma, J.; Li, N.; Guarnera, M.; Jiang, F. Quantification of Plasma miRNAs by Digital PCR for Cancer Diagnosis. *Biomark Insights* **2013**, *8*, 127–136, doi:10.4137/bmi.s13154.

59. Hennessey, P.T.; Sanford, T.; Choudhary, A.; Mydlarz, W.W.; Brown, D.; Adai, A.T.; Ochs, M.F.; Ahrendt, S.A.; Mambo, E.; Califano, J.A. Serum microRNA Biomarkers for Detection of Non-Small Cell Lung Cancer. *PLoS ONE* **2012**, *7*, doi:10.1371/journal.pone.0032307.

60. Heegaard, N.H.H.; Schetter, A.J.; Welsh, J.A.; Yoneda, M.; Bowman, E.D.; Harris, C.C. Circulating micro-RNA expression profiles in early stage nonsmall cell lung cancer. *Int. J. Cancer* **2012**, *130*, 1378–1386, doi:10.1002/ijc.26153.

61. Shen, J.; Todd, N.W.; Zhang, H.; Yu, L.; Lingxiao, X.; Mei, Y.P.; Guarnera, M.; Liao, J.P.; Chou, A.; Lu, C.L.; et al. Plasma microRNAs as potential biomarkers for non-small-cell lung cancer. *Lab. Invest.* **2011**, *91*, 579–587, doi:10.1038/labinvest.2010.194.

62. Roth, C.; Kasimir-Bauer, S.; Pantel, K.; Schwarzenbach, H. Screening for circulating nucleic acids and caspase activity in the peripheral blood as potential diagnostic tools in lung cancer. *Mol. Oncol.* **2011**, *5*, 281–291, doi:10.1016/j.molonc.2011.02.002.

63. Boeri, M.; Verri, C.; Conte, D.; Roz, L.; Modena, P.; Facchinetti, F.; Calabro, E.; Croce, C.M.; Pastorino, U.; Sozzi, G. MicroRNA signatures in tissues and plasma predict development and prognosis of computed tomography detected lung cancer. *Proc. Natl. Acad. Sci. USA* **2011**, *108*, 3713–3718, doi:10.1073/pnas.1100048108.

64. Bianchi, F.; Nicassio, F.; Marzi, M.; Belloni, E.; Dall'Olio, V.; Bernard, L.; Pelosi, G.; Maisonneuve, P.; Veronesi, G.; Di Fiore, P.P. A serum circulating miRNA diagnostic test to identify asymptomatic high-risk individuals with early stage lung cancer. *EMBO Mol. Med.* **2011**, *3*, 495–503, doi:10.1002/emmm.201100154.

| 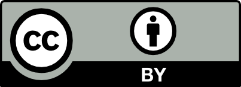 | © 2019 by the authors. Licensee MDPI, Basel, Switzerland. This article is an open access article distributed under the terms and conditions of the Creative Commons Attribution (CC BY) license (http://creativecommons.org/licenses/by/4.0/). |
| --- | --- |
